# Infection Augments Expression of Mechanosensing Piezo1 Channels in Amyloid Plaque-Reactive Astrocytes

**DOI:** 10.3389/fnagi.2018.00332

**Published:** 2018-10-22

**Authors:** María Velasco-Estevez, Myrthe Mampay, Hervé Boutin, Aisling Chaney, Peter Warn, Andrew Sharp, Ellie Burgess, Emad Moeendarbary, Kumlesh K. Dev, Graham K. Sheridan

**Affiliations:** ^1^Neuroimmulology & Neurotherapeutics Laboratory, School of Pharmacy and Biomolecular Sciences, University of Brighton, Brighton, United Kingdom; ^2^Drug Development, Department of Physiology, School of Medicine, Trinity College Dublin, Dublin, Ireland; ^3^Wolfson Molecular Imaging Centre, Faculty of Biology, Medicine and Health and Manchester Academic Health Sciences Centre, The University of Manchester, Manchester, United Kingdom; ^4^Department of Radiology, Stanford University, Stanford, CA, United States; ^5^Evotec (UK) Ltd., Manchester Science Park, Manchester, United Kingdom; ^6^Department of Biological Engineering, Massachusetts Institute of Technology, Cambridge, MA, United States; ^7^Department of Mechanical Engineering, University College London, London, United Kingdom

**Keywords:** Alzheimer’s disease, amyloid plaques, astrocytes, dentate gyrus, mechanosensitive ion channel, Piezo1, TgF344-AD rats, urinary tract infection

## Abstract

A defining pathophysiological hallmark of Alzheimer’s disease (AD) is the amyloid plaque; an extracellular deposit of aggregated fibrillar Aβ_1-42_ peptides. Amyloid plaques are hard, brittle structures scattered throughout the hippocampus and cerebral cortex and are thought to cause hyperphosphorylation of tau, neurofibrillary tangles, and progressive neurodegeneration. Reactive astrocytes and microglia envelop the exterior of amyloid plaques and infiltrate their inner core. Glia are highly mechanosensitive cells and can almost certainly sense the mismatch between the normally soft mechanical environment of the brain and very stiff amyloid plaques via mechanosensing ion channels. Piezo1, a non-selective cation channel, can translate extracellular mechanical forces to intracellular molecular signaling cascades through a process known as *mechanotransduction*. Here, we utilized an aging transgenic rat model of AD (TgF344-AD) to study expression of mechanosensing Piezo1 ion channels in amyloid plaque-reactive astrocytes. We found that Piezo1 is upregulated with age in the hippocampus and cortex of 18-month old wild-type rats. However, more striking increases in Piezo1 were measured in the hippocampus of TgF344-AD rats compared to age-matched wild-type controls. Interestingly, repeated urinary tract infections with *Escherichia coli* bacteria, a common comorbidity in elderly people with dementia, caused further elevations in Piezo1 channel expression in the hippocampus and cortex of TgF344-AD rats. Taken together, we report that aging and peripheral infection augment amyloid plaque-induced upregulation of mechanoresponsive ion channels, such as Piezo1, in astrocytes. Further research is required to investigate the role of astrocytic Piezo1 in the Alzheimer’s brain, whether modulating channel opening will protect or exacerbate the disease state, and most importantly, if Piezo1 could prove to be a novel drug target for age-related dementia.

## Introduction

The etiology of early-onset Alzheimer’s disease (AD) has a strong genetic component and mutations in genes that encode for amyloid precursor protein (APP), presenilin 1 (PSEN1), and presenilin 2 (PSEN2) give rise to a rare but severe form of familial dementia characterized by cortical and hippocampal atrophy and progressive cognitive decline (Saunders, 2001; Nacmias et al., 2014; Lanoiselee et al., 2017). The amyloid cascade hypothesis of AD (Hardy and Allsop, 1991) states that aberrant cleavage of APP by γ-secretase results in the accumulation of cytotoxic oligomeric amyloid-β_1-42_ (Aβ_1-42_) which aggregates and forms β-sheet fibrillar structures that, in turn, clump together and generate extracellular amyloid plaques (Lukiw, 2012; Morris et al., 2014). Amyloid plaques contribute to cortical and hippocampal neurodegeneration and neuroinflammation (Nobakht et al., 2011; Dansokho and Heneka, 2018), although extensive plaque pathology is not necessarily a good indicator of the clinical severity of dementia (Hyman et al., 1993). Structurally, amyloid plaques are very brittle and stiff (with a stiffness of ∼3 × 10^9^ Pa) (Smith et al., 2006), in contrast to brain tissue which we have previously shown to be very soft (with a stiffness of ∼200–500 Pa) (Moeendarbary et al., 2017). Therefore, neurons and glia surrounding the amyloid plaque experience a large perturbation to their mechanical microenvironment. Astrocytes, the most abundant glial cell type in the brain, respond to amyloid plaque accumulation by adopting a ‘reactive’ phenotype characterized by an increase in intermediate filament expression, e.g., GFAP (Wilhelmsson et al., 2006; Sofroniew, 2009), and synthesis of proinflammatory cytokines (Buffo et al., 2010; Sofroniew, 2014). We hypothesize that, in addition to biochemical signals, astrogliosis in the AD brain is triggered in part by a detectable increase in the stiffness of the mechanical microenvironment experienced by resting glial cells.

Neurons and glia are highly mechanosensitive (Previtera et al., 2010; Blumenthal et al., 2014) and detect changes in the stiffness of underlying cell culture substrates via stretch-activated membrane-spanning ion channels (Cox et al., 2016; Bavi et al., 2017). Microglial cells, for example, display durotaxis, i.e., they migrate toward stiffer areas when cultured on stiffness gradient hydrogels (Bollmann et al., 2015). Astrocytes grown on stiff hydrogels possess longer, thicker processes and appear much more reactive than those cultured on soft hydrogels (Moshayedi et al., 2010). Moreover, astrocytes *in vivo* respond to hard foreign body implants by releasing proinflammatory cytokines, such as Il-1β (Moshayedi et al., 2014). In AD, the build-up of extracellular amyloid plaques causes astrogliosis and, given the stiffness of plaques, we hypothesize that astrocyte reactivity is mediated partly by activation of mechanosensitive ion channels. Therefore, our aim here was to investigate cellular expression of mechanosensitive Piezo1 channels in response to amyloid plaque pathology in an aging rat model of AD.

To carry out this aim, we utilized the TgF344-AD rat model (Cohen et al., 2013) that overexpress human APP with the Swedish mutation (APP_SWE_) and mutated human PSEN1 (PSEN1ΔE9). In the healthy human brain, Piezo1 mRNA is expressed by neurons but is absent from astrocytes. Post-mortem analysis of AD brains, however, revealed that Piezo1 mRNA is down-regulated in neurons and up-regulated in astrocytes surrounding amyloid plaques (Satoh et al., 2006). Therefore, Piezo1 was previously known as Mib (membrane protein induced by beta-amyloid treatment). Piezo1 is a large protein (>2,500 amino acids) predicted to contain 30–40 putative transmembrane segments (Kamajaya et al., 2014), assembles as a ∼900 kDa homotrimer (Ge et al., 2015) and forms a non-selective calcium (Ca^2+^)-permeable cation channel (Gnanasambandam et al., 2015). Mechanosensitive ion channels translate external physical stimuli, such as shear stress (Ranade et al., 2014), pressure (Gottlieb and Sachs, 2012; Bae et al., 2015) and extracellular matrix (ECM)-associated traction forces (Pathak et al., 2014) to intracellular biochemical signals via mechanotransduction events (Martinac, 2004; Moeendarbary and Harris, 2014). Piezo1 mRNA is highly expressed in tissues subject to regular deformations or shear forces such as lungs, bladder, skin and vasculature endothelium (Coste et al., 2010; Ranade et al., 2014; Li et al., 2015). To date, however, very little is known about the role of Piezo1 in the CNS. We have recently shown that Piezo1 is involved in axon guidance of optic tract retinal ganglion cells (RGCs) in the developing *Xenopus laevis* brain (Koser et al., 2016). Here, we will investigate if glial cells can physically sense and respond to the accumulation of amyloid plaques by quantifying expression of astrocytic Piezo1 channels in the hippocampus and cerebral cortex of the aging TgF344-AD rat model of AD.

Because of their advanced age, elderly people are prone to co-morbidities and peripheral infections that cause low-grade systemic inflammation (Salles and Mégraud, 2007; Doulberis et al., 2018). Interestingly, peripheral infections increase older people’s risk of developing AD (Holmes et al., 2009; Tate et al., 2014) and reportedly exacerbate amyloid plaque deposition in the mouse brain (McManus et al., 2014), although the mechanisms underlying this crosstalk between the periphery and CNS is not well-understood. Although most transgenic animal models recapitulate many of the amyloid-related hallmarks of AD, it is important to use clinically-relevant animal models with at least one comorbidity, such as a peripheral infection, to investigate AD pathogenesis. Therefore, we investigated if a peripheral urinary tract infection (UTI) with *E. coli* bacteria, one of the most likely causes of infection in elderly people with dementia (Natalwala et al., 2008; Rowe and Juthani-Mehta, 2013), modifies astrocytic mechano-responsiveness to stiff extracellular amyloid deposits (Leyns and Holtzman, 2017). Our results suggest that restoring homeostatic mechanotransduction pathways and neuron/glial crosstalk around amyloid plaques may lead to the discovery of novel drug targets for neurodegeneration and AD.

## Materials and Methods

### Ethics Statement

All experiments involving animals and Schedule 1 protocols used to obtain brain tissue were approved by the Animal Welfare and Ethical Review Bodies (AWERB committees) of the University of Brighton and The University of Manchester, as well as the Animal Research Ethics Committee (AREC) in Trinity College Dublin. This study was conducted in accordance with the principles of the Basel Declaration and adhered to the legislation detailed in the United Kingdom Animals (Scientific Procedures) Act 1986 Amendment Regulations (SI 2012/3039). All efforts were taken to maximize animal welfare conditions and to reduce the number of animals used in accordance with the European Communities Council Directive of September 20^th^, 2010 (2010/63/EU).

### Mouse Neuron Cultures

Primary neuron cultures were prepared from the cerebral cortical tissue of male and female postnatal day 0 or 1 (P0 or P1) C57BL/6 mice bred in the BioResources Unit (BRU) of University of Brighton. Briefly, cortices were dissected following decapitation and collected in Ca^2+^- and Mg^2+^-free Hank’s balanced salt solution (HBSS) (Thermo Fisher Scientific, 14185052). Cells were dissociated using Papain [20 U/mL] (Worthington, 3119) in Ca^2+^- and Mg-free HBSS supplemented with L-cysteine [1 mM] (Sigma, 168149), EDTA [0.5 mM] (Thermo Fisher Scientific, 15575020) and DNAse I solution [0.125 U/mL] (Thermo Fisher Scientific, 90083). Dissociation was inhibited using Type II-O ovomucoid trypsin inhibitor [0.1%] (Sigma, T9253) in HBSS with bovine serum albumin [0.1%] (Sigma, A9647) and D-APV [D-(-)-2-Amino-5-phosphonopentanoic acid] [50 μM] (Abcam, ab120003). A single-cell suspension was created by gentle manual trituration using fire-polished Pasteur pipettes. Cells were plated on Poly-D-Lysine (Millipore, A-003-E) coated coverslips in 24-well plates [6 × 10^4^ cells/well] with serum-free Neurobasal-A medium (Life Technologies, 10888-022) supplemented with 2% B27 (Thermo Fisher Scientific, 17504044) and L-glutamic acid [25 μM] (Sigma, G1251). Primary neuron cultures were maintained at 37°C and 5% CO_2_ in a humidified incubator and after 1 day *in vitro*, half of the plating medium was replaced with maintenance medium (without L-glutamic acid) composed of Neurobasal-A medium supplemented with 2% B27, 2 mM GlutaMAX^TM^ (Gibco, 35050061), and 1% penicillin/streptomycin (Thermo Fisher Scientific, 15140122). This was repeated every 3 days, i.e., 50% of the cell culture medium was replenished with fresh maintenance medium. After 14 days in culture, cells were fixed in ice-cold formalin solution (10%) for immunofluorescent labeling.

### Mouse Astrocyte Cultures

Mixed glial cell cultures were prepared from the cerebral cortical tissue of male and female P1 wild-type C57BL/6 mice bred in the BioResources Units of Trinity College Dublin and University of Brighton, following previously described protocols (O’Sullivan et al., 2017, 2018). Briefly, following decapitation the cortices were dissected with sterile forceps and placed in HBSS (Gibco, 14025-050). Cortical tissue was cross-chopped with a sterile scalpel and then placed in pre-warmed Dulbecco’s modified Eagle’s medium/F12 (DMEM/F12, Hyclone, SH30023) supplemented with 10% FBS (Labtech; FB-1090) and 1% penicillin/streptomycin (Sigma, P4333) and gently triturated until a clear solution was obtained. The resulting solution was passed through a cell strainer (40 μm) and plated into separate T75 flasks. Cells were cultured at 37°C and 5% CO_2_ in a humidified incubator and grown in the supplemented DMEM/F12 media previously described. After 12 days in culture, microglial cells were separated from mouse astrocytes by placing them on a rotating shaker for 2 h at 37°C. The supernatant, with detached microglia, was removed and the enriched astrocyte cultures were trypsinised with 0.5% trypsin for 7 minutes (min) and plated accordingly. Mouse astrocyte purity was >97%, as previously described (Healy et al., 2013).

### Aβ_1-42_ Conditioned Microglial Media

Microglial cultures were prepared from the cerebral cortical tissue of male and female P1 wild-type C57BL/6 mice bred in the BioResources Unit of Trinity College Dublin, as previously described (Dempsey et al., 2017). Briefly, after 48 h in culture, microglia were treated with 10 μM amyloid-β_1-42_ peptide (Invitrogen, United Kingdom) for 24 h. Aβ_1-42_ conditioned microglial media (CMM) was collected and used to treat the enriched astrocyte cultures described above.

### Immunocytochemistry

After each biochemical treatment, mouse astrocytes were fixed in 10% formalin solution (Sigma, F1635) for 5 min on ice. Cells were blocked overnight at 4°C in blocking solution, i.e., 1% BSA + 0.1% Triton X-100. Primary antibodies included chicken anti-neurofilament H (NFH; Millipore, AB5539, RRID: AB_177520) 1:1000 dilution, rabbit anti-GFAP (Abcam, ab7260, RRID: AB_305808) 1:1000 dilution, goat anti-PIEZO1 (N-15; Santa Cruz, sc-164319, RRID: AB_10842990) 1:500 dilution, and rabbit anti-FAM38A (Abcam, ab128245, RRID: AB_11143245) 1:500 dilution in blocking solution, were used and incubated overnight at 4°C. Cells were washed with PBS + 0.1% Triton X-100 for 5 min, three times. Incubations with secondary antibodies were performed in the dark at 22 ± 2°C for an hour. In the case of rabbit anti-FAM38A, it was also incubated with biotinylated goat anti-rabbit antibody (Thermo Fisher, A24535, RRID: AB_2536003) and avidin Alexa 488 conjugate (Life Technologies, A21370) to amplify the fluorescent signal. After washes, cells were coverslipped and mounted on a microscope slide (Clarity, C361) with antifade reagent (Thermo Fisher S36936). Imaging was performed using a Leica SP8 confocal microscope.

### Western Blotting

Mouse astrocyte samples were obtained by scraping the cells in radioimmunoprecipitation assay buffer (RIPA) containing 150 mM NaCl (Sigma, A3014), 1% Triton-X (Sigma, T8787), 0.1% SDS (Fisher, S/5200/53) and 50 mM Tris pH 8.0 (Fisher, T/3710/60). Samples were sonicated three times for 10 s at 20% amplitude using a vibracell VCX 130 (Sonics, United States). Briefly, samples were mixed in 1:1 dilution with Laemmli sample buffer 2× (BioRad, 161-0737) and boiled at 95°C for 5 min. They were run in a 6% polyacrylamide separating gel with 4% stacking gel (Applichem Panreac, A1672) in running buffer at constant voltage 120 V, followed by wet transfer to a PVDF (Millipore, IPVH00010) for 3 h at constant 70 V on ice. Blocking of the membrane was done with 5% BSA (Santa Cruz, sc-2323) in 0.05% PBS/Tween 20 for an hour at 22 ± 2°C. Incubation with primary antibody rabbit anti-FAM38A (Abcam, ab128245, RRID: AB_11143245) or goat anti-PIEZO1 (Santa Cruz, sc-164319, RRID: AB_10842990) was performed at a 1:500 dilution for 48 h at 4°C. The membrane was washed and incubated with secondary antibody HRP-conjugated goat anti-rabbit (GE Healthcare, NA934, RRID: AB_772206) or HRP-conjugated rabbit anti-goat (Sigma, A4174, RRID: AB_258138), respectively, at 1:5000 dilution for 2 h at 22°C. Following further washes, membranes were developed using chemiluminescent HRP substrate (Millipore, WBKLS0500) in a Fujifilm LAS-3000 (Fujifilm, Japan).

### Wild-Type Rats

Female Lister hooded rats (Harlan Laboratories, United Kingdom) were socially housed in groups of four and given *ad libitum* access to food and water. The holding room was kept on a 12 h light/dark cycle at 22 ± 2°C. Postnatal day 37 (5-weeks) rats were sacrificed by pentobarbital overdose (intraperitoneal injection) and death was confirmed by decapitation. Immunohistochemical experiments were conducted using post-mortem brain tissue. All experiments on animal tissue were performed in accordance with the United Kingdom Animals (Scientific Procedures) Act 1986 Amendment Regulations (SI 2012/3039).

### TgF344-AD Rats

Two male and female WT Fisher and TgF344-AD (TG) rats with the APP_SWE_ and PSEN1ΔE9 mutations were purchased from Prof T. Town laboratory (University of Southern California, United States) and were set-up as breeding pairs in-house at the Biological Services Unit (BSU) in The University of Manchester, United Kingdom. TG rats and WT littermates were randomly split into four groups, i.e., WT and TG non-infected (WT/TG) and infected (iWT/iTG), bearing in mind that the UTI needed to be performed on the entire box of rats. The number of rats per group (n) were as follows; 12m WT (*n* = 5); 18m WT (*n* = 5); 12m TG (*n* = 6); 18m TG (*n* = 6); 12m iWT (*n* = 5); 18m iWT (*n* = 5); 12m iTG (*n* = 6); 18m iTG (*n* = 6). All animals were housed in groups of 2–4 in cages with individual ventilation, environmental enrichment and constant access to food and water. A 12 h light/dark cycle was used, with light from 7 am until 7 pm. All experiments were carried out following internal ethical review and approval by the AWERB committee of The University of Manchester.

### Urinary Tract Infection

A UTI was administered to the iWT and iTG groups at 7–8, 10–11, 13–14, and 16–17 months of age using *Escherichia coli* (*E. coli*) UTI89. For infections at 7–8 and 10–11 months of age, a concentration of 1.3 × 10^9^ colony forming units/mL (cfu/mL) was used, and for infections at 13–14 and 16–17 months of age, a concentration of 2.6 × 10^9^ cfu/mL was used. Therefore, before sacrifice, 12-month rats received two rounds of infection and 18-month rats received four rounds. Prior to infection, stocks of *E. coli* were stored as 1.02 × 10^11^ cfu/mL in 15% glycerol and were rapidly thawed and brought to the correct concentration using sterile PBS. Rats were anesthetized and infected with 0.1 mL of *E. coli* UTI89 by inserting a catheter (polyethylene tubing covering 30-G hypodermic needles) into the urethra and injecting the inoculum into the bladder. Prior to infection, buprenorphine (0.03 mg/kg) was administered and the animals’ bladders were emptied by gently pressing on the abdomen to release any bladder content. Infected animals were placed into clean cages and were monitored for 5 days for signs of illness. Urine samples were taken 2 and 5 days post-infection to confirm infection levels. Urine was plated on CLED agar plates and incubated at 37°C for 18–24 h after which colony forming unit counts were performed and scored according to the following classification system: 0 = Zero colonies; 0.5 = Scanty growth (<5 cfu); 2 = Scanty growth (<20 cfu); 3 = moderate growth (>20 cfu); 4 = confluent growth. Non-infected rats tested for infection all scored ≤ 0.5.

### Tissue Sectioning

Coronal slices of Lister hooded rat brains (3–4 mm thick) were fixed in 10% formalin solution for 8 h at 4°C and then transferred to 30% sucrose solution overnight (4°C) for cryoprotection. Brain slices were then covered in OCT (Optimal Cutting Temperature compound) and frozen and stored at -80°C. The slices were cryosectioned (14 μm thick) coronally at approximately -3.0 and -10.8 mm with respect to Bregma (Paxinos and Watson, 2014) in order to reveal the dorsal hippocampus and cerebellum regions, respectively. Cryosections were adhered to glass slides coated with poly-L-lysine solution [0.1% (w/v) in H_2_O, Sigma]. Sections were permeabilized using a solution of 0.1% Triton^TM^ X-100 (Sigma) in PBS for 25 min.

TgF344-AD and WT rats were sacrificed using an isoflurane overdose, confirmed by cervical dislocation. The brains were collected, snap frozen using isopentane on dry ice and stored at -80°C. Sagittal brain sections (20 μm thick) between 1 and 3.36 mm lateral to Bregma were taken using a cryostat (Leica CM3050s, Leica Biosystems Nussloch GmbH, Germany) and stored at -80°C. Prior to immunofluorescence, frozen sections were allowed to air dry at 22 ± 2°C for 20 min before fixation in 70% ethanol for 30 min. They were permeabilized in 0.2% Triton-X/PBS for 30 min, followed by several PBS washes, and then blocked with 5% BSA/PBS for 1.5 h at 22 ± 2°C.

### Immunohistochemistry

Sections from WT Fisher and TgF344-AD rats were incubated overnight at 22 ± 2°C with the following primary antibodies, (1) 1:500 dilution of goat anti-PIEZO1 (N-15; Santa Cruz, sc-164319, RRID: AB_10842990), an affinity-purified goat polyclonal antibody raised against a peptide mapping to the N-terminus of PIEZO1 of human origin; (2) 1:1000 dilution of rabbit anti-amyloid β_1-42_ (mOC98, Abcam, ab201061), a conformation-specific antibody that recognizes a discontinuous epitope of Aβ that maps to segments AEFRHD and EDVGSNK; (3) 1:1000 dilution of chicken anti-GFAP (Abcam, ab4674, RRID: AB_304558). Slides were then washed in PBS and incubated for 4 h at 22°C with the following secondary antibodies, (1) donkey anti-goat Alexa 488 (Abcam, ab150133), (2) donkey anti-rabbit 555 (Sigma, SAB4600061), (3) donkey anti-chicken IgY (H + L) CF^TM^ 633 (Sigma, SAB4600127). Finally, slides were washed and coverslipped using ProLong^®^ Gold antifade mounting medium with DAPI (Life Technologies, United Kingdom, P36935).

### Microscopy and Image Analysis

All images used to quantify Piezo1 expression in sagittal cryosections were captured using an Axio Scan.Z1 slide scanner (Zeiss, Germany) with a 20× magnification objective (Plan-Apochromat). Individual images were montaged together automatically in ZEN software (Zeiss, Germany) to reconstruct a single image of the whole brain section. For quantitative fluorescence intensity analysis, all images were captured in a single uninterrupted run (∼50 h imaging time) and uniform microscope settings were maintained throughout the session. The images were exported as 8-bit tif files for Piezo1 fluorescence intensity quantification. Image analysis was conducted using the software package FIJI^1^. Briefly, Piezo1 expression was quantified by manually selecting 10 regions of interest (ROI) for each brain structure and calculating the average fluorescence intensity within each ROI. Two or three brain sections from 5 or 6 different animals per age group/genotype were analyzed (*n* = 5–6). More specifically, the number of rats per group were as follows; 12m WT (*n* = 5); 18m WT (*n* = 5); 12m TG (*n* = 6); 18m TG (*n* = 6); 12m iWT (*n* = 5); 18m iWT (*n* = 5); 12m iTG (*n* = 6); 18m iTG (*n* = 6). Therefore, 100–180 fluorescence intensity (ROI) measurements were captured depending on the age and genotype of the rat. Piezo1 fluorescence intensities were normalized to the 12m WT value for each region and the percentage (%) change relative to the 12m WT group is represented in each bar graph. To quantify Piezo1 expression in amyloid plaque-reactive astrocytes versus astrocytes located at least 200 μm away from any noticeable amyloid deposits, 25 plaques from the frontal cortex of 18m TG rat brains were randomly selected for analysis. For each image (25 in total) Piezo1 fluorescence intensity was quantified in six astrocytes within the plaque core and in six astrocytes ∼200–500 μm away from the plaque. The average Piezo1 fluorescence intensity values for astrocytes located “outside” the plaque versus “inside” the plaque were calculated for each image, i.e., 150 astrocytes per group were analyzed.

### Statistical Analysis

All statistical analysis was performed using GraphPad^®^ Prism 7 (RRID: SCR_015807). Assessment of the normality of data was carried out using column statistics with D’Agostino analysis before any other statistical test was performed. For Western blot assays, repeated measures analysis of variance (ANOVA) tests were performed because data in every experiment was matched. Holm-Sidak multiple comparisons *post hoc* tests were run in conjunction with one-way ANOVAs and all groups were compared with one another. Data are presented as the mean ± standard error of the mean (SEM). To analyze changes in Piezo1 fluorescence intensities *in vivo*, where three independent variables (i.e., age, genotype, and infection) were present, a three-way ANOVA was performed on the raw fluorescence values, followed by a Holm-Sidak multiple comparisons *post hoc* test. Changes in Piezo1 expression *in vivo* were only considered significant when they passed two criteria, i.e., the relative percentage (%) change was > 20% and the *p*-value < 0.01. To analyze differences in average astrocytic Piezo1 expression outside versus inside amyloid plaques in the frontal cortex of 18m TG rat brains, a two-tailed paired *t*-test was performed. Finally, to correlate Piezo1 expression in the DG with that of GFAP and Aβ_1-42_ fluorescence intensities, Pearson *r*-values were calculated using the raw fluorescence intensity values. A correlation was only deemed significant if *r* > 0.5 and *p* < 0.0001.

## Results

### Mechanosensitive Piezo1 Channel Expression in the Juvenile and Adult Rat Brain

**FIGURE 1:**
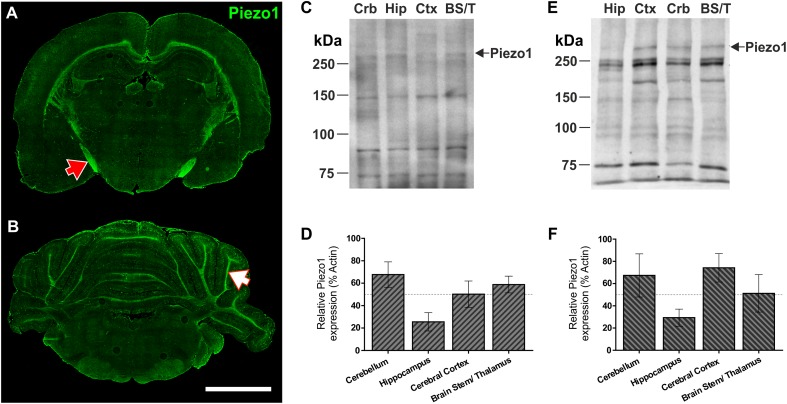
Piezo1 channels are highly expressed by neurons in the optic tract, corpus callosum, and arbor vitae of the cerebellum. Coronal sections of the 5-week old rat brain were cut at –3.3 mm **(A)** and –10.8 mm **(B)** with respect to Bregma and immunofluorescently stained for Piezo1 (N-terminal antibody) (*n* = 4), scale bar = 4,000 μm. The red arrow points to the optic tract in the right hemisphere and the white arrow points to the arbor vitae of the Ansiform lobule Crus 1 of the cerebellum. 5-week old rat brains were also dissected into four parts for Western blotting (WB), i.e., cerebellum (Crb), hippocampus (Hip), cerebral cortex (Ctx) and brainstem/thalamus (BS/T). WB were run for Piezo1 using an N-terminal binding antibody **(C,D)** from Santa Cruz (*n* = 3) and a C-terminal binding antibody **(E,F)** from Abcam (*n* = 3). The hippocampus consistently showed the lowest levels of Piezo1 protein expression.

To characterize Piezo1 channel expression in distinct regions of the young rat brain, coronal sections were cut at -3.0 and -10.8 mm with respect to Bregma revealing hippocampal (Figure 1A) and cerebellar (Figure 1B) structures. In agreement with our previous findings in the brain of embryonic *Xenopus laevis* (Koser et al., 2016), the juvenile rat optic tract expresses relatively high levels of Piezo1 channels (Figure 1A, red arrow). Moreover, axonal tracts of the corpus callosum, hippocampal fimbriae, and arbor vitae of the cerebellum (Figure 1B, white arrow) express high levels of Piezo1 channels. In contrast, the molecular and granule layers of the cerebellum, and non-neuronal areas of the hippocampus, express much fewer Piezo1 channels (Figures 1A,B). These regional differences were confirmed by Western blot (WB) using two distinct antibodies, i.e., one which binds the N-terminus of Piezo1 (Santa Cruz; Figures 1C,D) and one that binds the C-terminus (Abcam; Figures 1E,F). For WB, the brains of juvenile rats were dissected into four areas, i.e., cerebellum (Crb), hippocampus (Hip), cerebral cortex (Ctx), and brain stem/thalamus (BS/T) combined. There were no statistically significant differences in Piezo1 expression between brain structures (repeated measures one-way ANOVA, *p* > 0.05). However, the hippocampus consistently displayed lower levels of Piezo1, both with immunofluorescence and WB techniques.

**FIGURE 2:**
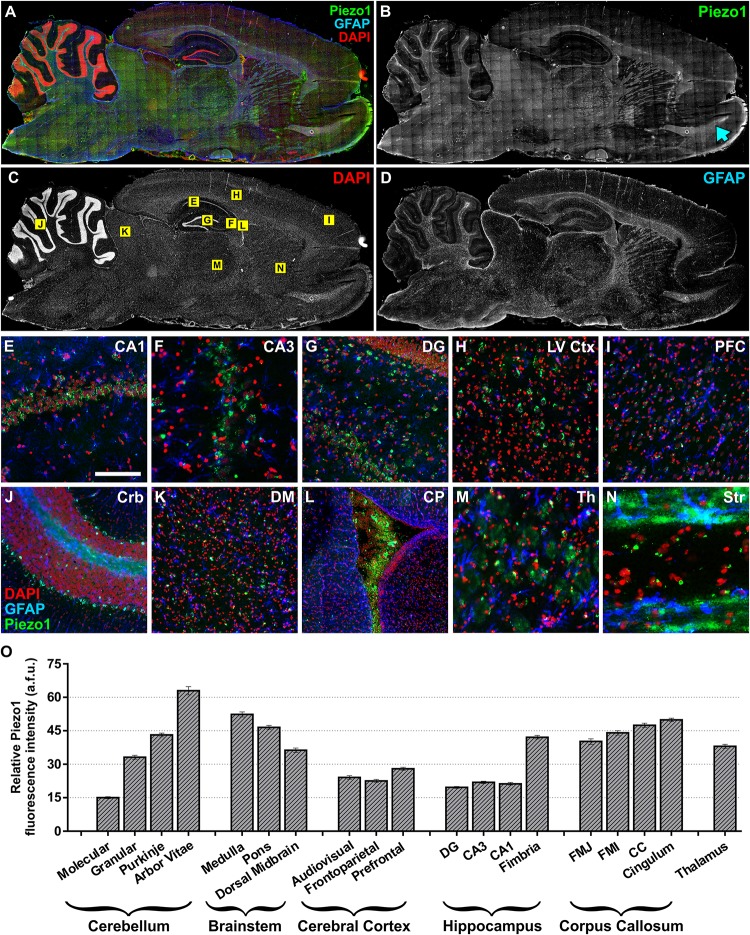
Piezo1 channel expression in the adult rat brain. **(A)** The brains of adult (12 month) Fisher rats were sectioned in the sagittal plane (lateral –1.9 mm) and stained for **(B)** Piezo1 (N-terminal antibody), **(C)** DAPI, and **(D)** GFAP (*n* = 5). Images were captured using a 20× magnification objective on an Axio Scan.Z1 slide scanner (Zeiss, Germany) and montaged in ZEN software. 20× magnification images of the hippocampal **(E)** CA1 (scale bar = 150 μm), **(F)** CA3 and **(G)** dentate gyrus (DG) show that Piezo1 expression is greater in pyramidal and granule neurons compared to GFAP-positive astrocytes. In the cerebral cortex, **(H)** layer V pyramidal neurons (LV Ctx) appear to express more Piezo1 channels than **(I)** prefrontal cortical (PFC) neurons. In the cerebellum **(J)**, Purkinje neuron cell bodies and axons (arbor vitae) express higher levels of Piezo1 compared to neurons in the granule and molecular cell layers. **(K)** The dorsal midbrain (DM) expresses moderate levels of Piezo1, whereas **(L)** epithelial cells of the choroid plexus (CP) express greater amounts of Piezo1 protein. It was also noted that white matter tracts in **(M)** the thalamus (Th) and **(N)** striatum (Str) express more Piezo1 than gray matter areas of these brain regions. The areas depicted in images **(E–N)** are shown as yellow boxes in **(C)**. Relative fluorescence intensity (arbitrary fluorescence units, a.f.u.) of Piezo1 staining in each brain region was quantified **(O)**. Axonal tracts including the cerebellar arbor vitae, hippocampal fimbriae, cingulum and corpus callosum, as well as the medulla and pons of the brainstem, had the highest expression of Piezo1 protein.

Next, sagittal sections from adult (12 months) rat brains were imaged to characterize Piezo1 localisation in several other structures along the anterior–posterior axis (Figures 2A–D). Similar to juvenile rats, Piezo1 was highly expressed along axonal tracts of the corpus callosum and cerebellar arbor vitae as well as on Purkinje cell bodies (Figure 2J), medulla, pons, thalamus (Figure 2M) as well as on Purkinje cell bodies (Figure 2J), medulla, pons, thalamus (Figure 2M), striatum (Figure 2N) and the rostral migratory stream which links the subventricular zone to the olfactory bulb (Figure 2B, blue arrow). Areas of low Piezo1 expression included the dorsal midbrain (Figure 2K) and non-neuronal regions of the hippocampus. Pyramidal and granule neurons of the CA1 (Figure 2E), CA3 (Figure 2F), and dentate gyrus (Figure 2G), however, expressed higher levels of Piezo1. Interestingly, layer V cortical cells (Figure 2H) expressed more Piezo1 channels than prefrontal cortical cells (Figure 2I). It was also noted that epithelial cells of the choroid plexus within the lateral ventricle (Figure 2L) express relatively high levels of Piezo1 protein.

### Reactive Astrocytes Surrounding Amyloid Plaques Upregulate Piezo1

**FIGURE 3:**
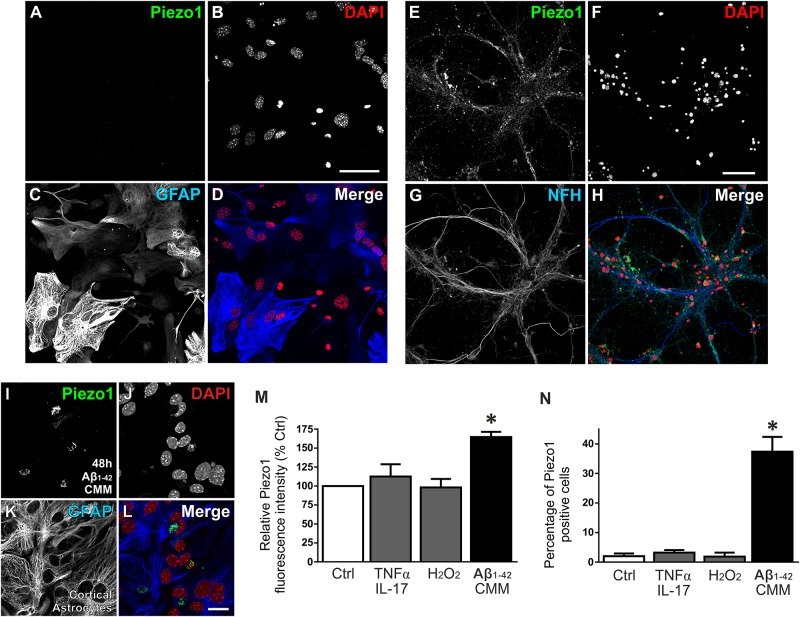
Aβ_1-42_ CMM upregulates Piezo1 expression in cortical astrocyte cultures. To check cellular localisation of Piezo1 in the brain, enriched astrocyte cultures **(A–D)** and cortical neuronal cultures **(E–H)** from newborn mice were stained for **(A,E)** Piezo1 (N-terminal antibody) and **(C)** GFAP or **(G)** neurofilament H (NFH), respectively (*n* = 3). Piezo1 co-localized with NFH staining in neurons **(H)** but was almost completely absent from astrocytes under basal conditions **(D)**. To investigate if neuroinflammatory stimuli can induce Piezo1 expression in astrocytes, mouse microglia were exposed to human Aβ_1-42_ (Invitrogen, United Kingdom) for 24 h and the inflammatory media was collected. Enriched mouse astrocyte cultures were then exposed to this Aβ_1-42_ CMM for 48 h. Astrocytes were immunofluorescently stained for **(I)** Piezo1 (C-terminus antibody), **(J)** DAPI, **(K)** GFAP, and **(L)** is the merge of each channel. Scale bar = 20 μm. Exposure to Aβ_1-42_ CMM upregulated Piezo1 protein expression in approximately 30–40% of astrocytes **(M,N)**. Astrocytes were also treated for 24 h with either 5 μM hydrogen peroxide (H_2_O_2_) or a cytokine cocktail consisting of TNF-α (10 ng/mL) and IL17A (250 ng/mL). Neither H_2_O_2_ nor TNF-α/IL17A increased Piezo1 expression indicating that Piezo1 upregulation is not a common response to all cell stressors but specific to certain inflammatory stimuli including Aβ_1-42_ and factors released by reactive amyloid-stimulated microglia. Data are represented as mean ± SEM (*n* = 8). A repeated measures one-way ANOVA with Holm-Sidak *post hoc* test was performed. ^∗^Represents a statistically significant difference (*p* < 0.05) from control (Ctrl), TNF-α/IL17A, and H_2_O_2_ treatment groups.

The pattern of expression in the rat brain suggested that Piezo1 is predominantly localized to neurons, in agreement with human studies of mRNA (Satoh et al., 2006). To examine if Piezo1 protein expression in mouse brain *in situ* matched that of the rat brain at the same age, we sectioned and immunofluorescently-labeled juvenile (5 weeks) mouse brains for Piezo1 and observed a strikingly similar expression pattern to the young rat brain; a result that matches the highly conserved amino acid sequence between mouse and rat Piezo1^2^. It was noted that Piezo1 expression in mouse brain also appeared predominantly neuronal (*data not shown*). To confirm cellular localisation, primary neuron cultures and enriched astrocyte cultures (∼97% pure) were prepared from newborn C57BL/6 mice and double-stained for either Piezo1 and GFAP (Figures 3A–D) or Piezo1 and neurofilament H (NFH; Figures 3E–H). Piezo1 co-localized with neurofilament H in mouse cortical neurons but was almost completely absent from primary astrocyte cultures under basal conditions.

To investigate if neuroinflammatory cell stressors are capable of upregulating Piezo1 channels in astrocytes, we exposed astrocytes to 5 μM hydrogen peroxide (H_2_O_2_) or to a cytokine cocktail consisting of TNF-α (10 ng/mL) and IL17A (250 ng/mL) for 24 h. Neither stressor induced Piezo1 expression in astrocytes (Figures 3M,N). Satoh et al. (2006), however, have reported previously that Piezo1 mRNA (then known as membrane protein induced by beta amyloid, Mib), is upregulated in astrocytes surrounding amyloid plaques in human post-mortem brain tissue. To examine if mouse astrocytes in culture can also be forced to express Piezo1 under similar pathophysiological conditions, we first treated mouse microglial cells with 10 μM amyloid beta-42 (Aβ_1-42_; Invitrogen, United Kingdom) for 24 h and collected the inflammatory cell culture media. Mouse astrocytes were then incubated with Aβ_1-42_ CMM for 48 h and stained for Piezo1 channels (Figures 3I–L). Aβ_1-42_ CMM induced Piezo1 expression in 30–40% of cortical astrocytes (Figures 3I,M,N).

**FIGURE 4:**
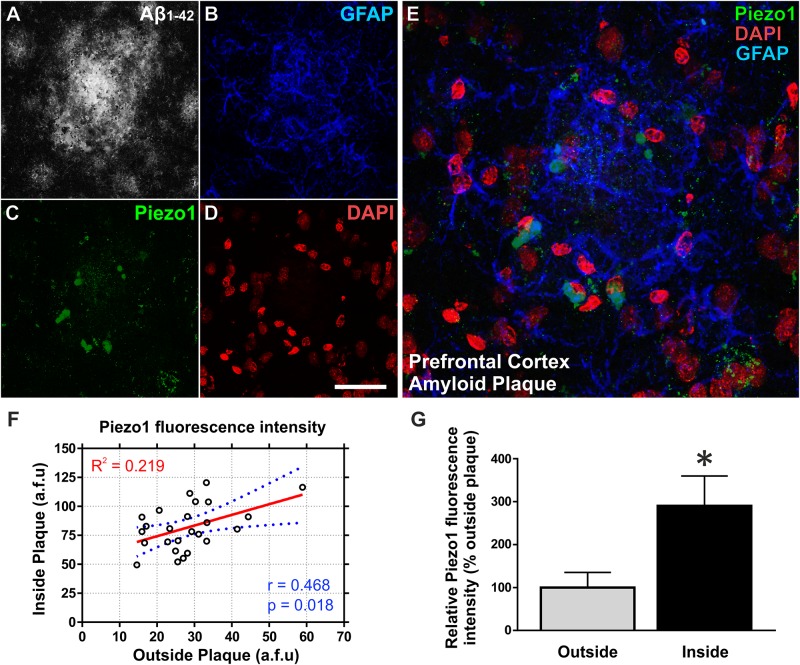
Aβ_1-42_ pathology *in vivo* upregulates Piezo1 expression in astrocytes surrounding amyloid plaques. 18-Month old TgF344-AD rat brains were immunofluorescently stained for **(A)** conformation-specific Aβ_1-42_, **(B)** GFAP, **(C)** Piezo1 (N-terminal antibody), and **(D)** DAPI. Scale bar = 40 μm. **(E)** High magnification (100× objective) z-stack projections of prefrontal cortex amyloid plaques reveal an upregulation of GFAP in reactive astrocytes surrounding the amyloid plaque. Piezo1 protein is also upregulated in a proportion of astrocytes in and around the core of the plaque. Piezo1 appears localized predominantly to the perinuclear compartment of Aβ_1-42_ reactive astrocytes. **(F)** Astrocytic Piezo1 fluorescence intensity was calculated for astrocytes inside the amyloid plaque versus astrocytes located 200–500 μm away from any noticeable plaque deposits. Piezo1 fluorescence intensity was averaged for six astrocytes “inside” and six astrocytes “outside” the plaque and 25 images from the frontal cortex of 18 m TG rats (*n* = 6) were analyzed. There was no correlation (Pearson r = 0.468) between Piezo1 expression inside versus outside amyloid plaques. **(G)** However, on average there was a 190% increase in Piezo1 expression in astrocytes within the plaque core (Inside) versus astrocytes at least 200 μm away from the edge of any amyloid plaque (Outside). A two-tailed paired *t*-test was performed. ^∗^Represents a statistically significant difference (*p* < 0.001) inside versus outside of the plaque.

Next, we investigated if upregulation of Piezo1 channels is also evident *in vivo* in an aging rat model of Alzheimer’s disease pathology (TgF344-AD). To achieve this, the brains of 18-month old TgF344-AD (18m TG) rats were sectioned in the sagittal plane and triple-immunolabeled for Piezo1, conformation-specific Aβ_1-42_ [mOC98], and GFAP. High magnification (100×) z-stack projections clearly demonstrate an upregulation of Piezo1 in reactive astrocytes surrounding prefrontal cortical amyloid plaques (Figures 4A–E). On average, there was a threefold upregulation in Piezo1 expression in amyloid plaque-reactive astrocytes compared to astrocytes located at least 200 μm away from any plaque in the prefrontal cortex (Figures 4F,G). One must remember, however, that GFAP staining only covers ∼15% of the total volume of astrocytes (Sun and Jakobs, 2012) and therefore, the reported percentage of cells expressing Piezo1 might be somewhat of an underestimation. Interestingly, *in vivo* Piezo1 expression closely matches our *in vitro* results following incubation of enriched astrocyte cultures with Aβ_1-42_ CMM, i.e., only a proportion of astrocytes (∼30%) express Piezo1 channels and the expression appears perinuclear in reactive astrocytes *in vivo*.

### Aging and Amyloid Plaque Pathology Increase Piezo1 Expression in Anterior Cortical Regions

**FIGURE 5:**
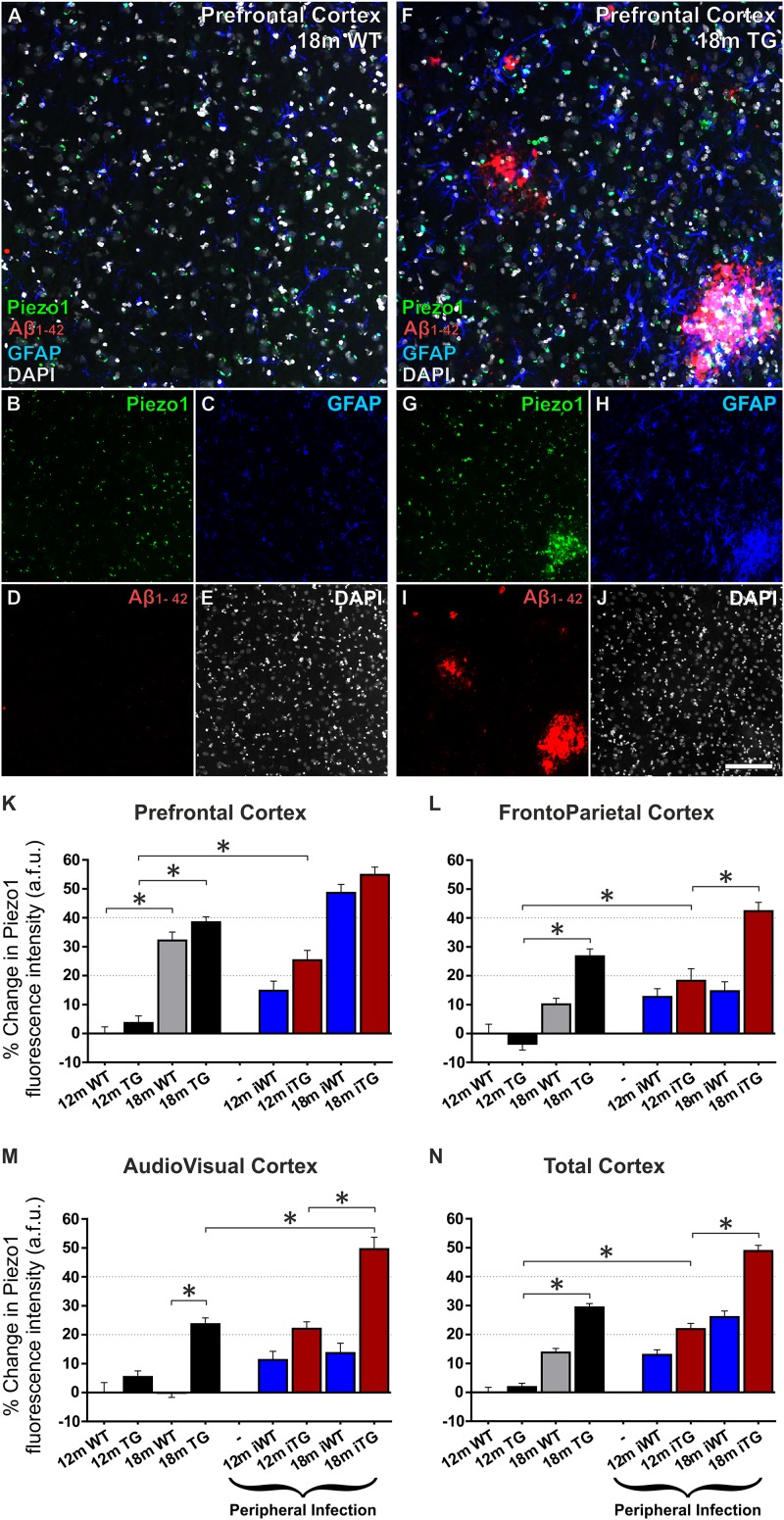
Mechanosensing Piezo1 channel expression increases in the cerebral cortex of TgF344-AD rats with a urinary tract infection (UTI). Sagittal sections (lateral –1.9 mm) of wild-type (WT) Fisher and TgF344-AD rat brains were immunofluorescently stained for Piezo1 (N-terminal antibody), conformation-specific Aβ_1-42_, GFAP, and DAPI. Shown are representative images of the prefrontal cortex of an 18-month WT rat **(A–E)** and an 18-month TgF344-AD (18 m TG) **(F–J)**. Scale bar = 150 μm. Elevations in Piezo1 channel expression with age, Alzheimer’s disease (AD) pathology and peripheral infection were measured in three distinct areas of the cerebral cortex, i.e., the prefrontal cortex **(K)**, the frontoparietal cortex **(L)**, and the audiovisual cortex **(M)**. In addition, Piezo1 expression was averaged over all three regions to illustrate changes in channel expression in the total cerebral cortex **(N)**. Data were normalized to 12 month wild-type (12m WT) values for each cortical region and are represented as the mean ± SEM of the percentage change from the 12m WT groups. Three-way ANOVAs with Holm-Sidak *post hoc* tests were performed to test for interactions between age, genotype, and peripheral infection. ^∗^Represents a change > 20% and a *p*-value < 0.01. There were 5–6 rats per group and 2–3 brain sections per animal. Ten regions of interest (ROI) were analyzed per cortical region per section, i.e., 100–180 ROIs analyzed per group.

We next characterized spatiotemporal changes in Piezo1 expression in different areas of the cerebral cortex in 12- and 18-month old wild-type (WT) and TgF344-AD (TG) rats. Low magnification (20× montage) images of the prefrontal cortex reveal an upregulation of Piezo1 and GFAP around amyloid plaques (Figures 5A–J). Piezo1 expression in the prefrontal cortex increased 32 ± 3% from 12 to 18 months of age in WT rats (Figure 5K; *p* < 0.0001). However, frontoparietal (Figure 5L) and audiovisual (Figure 5M) cortical regions did not display any significant changes in Piezo1 expression in WT rats from 12 to 18 months. Piezo1 channel expression also increased in the prefrontal (34 ± 2%) and frontoparietal (31 ± 2%) cortices of TG rats from 12- to 18-months of age but not in the audiovisual area (17 ± 2%). Taken together, both aging and amyloid plaque pathology trigger increases in Piezo1 channel expression, particularly in the prefrontal cortex, of TgF344-AD rats (Figures 5K–N).

### Peripheral Bacterial Infection Enhances Piezo1 Expression in the Cerebral Cortex of TgF344-AD Rats

Twelve month old TgF344-AD rats had previously been exposed to a UTI (*E. coli*) at 8 and 11 months of age (iTG). This bacterial infection caused a significant upregulation of Piezo1 channels in the prefrontal (21 ± 3%; Figure 5K) and frontoparietal (23 ± 3%; Figure 5L) cortices of 12-month old iTG rats, but not the audiovisual cortex (16 ± 2%; Figure 5M). In contrast, in older 18-month iTG rats that had been infected with *E. coli* at 8, 11, 14, and 17 months of age, no changes in Piezo1 channel expression were measured in prefrontal (12 ± 2%; Figure 5K) or frontoparietal (12 ± 3%; Figure 5L) cortices compared to non-infected TgF344-AD counterparts. However, Piezo1 expression in the audiovisual cortex did display significant upregulations (21 ± 3%; Figure 5M) in 18-month iTG rats in response to repeated peripheral infections. Interestingly, peripheral *E. coli* infections did not increase Piezo1 expression in the prefrontal cortex of WT rats at 12 months (15 ± 3%) nor at 18 months (12 ± 3%) of age compared to WT controls. Taken together, our results indicate that repeated UTI enhances Piezo1 expression in the cerebral cortex of TgF344-AD rats over and above that seen in response to amyloid plaques alone (Figure 5N).

### Piezo1 Upregulation in the Hippocampus of TgF344-AD Rats Precedes Changes in the Cerebral Cortex

**FIGURE 6:**
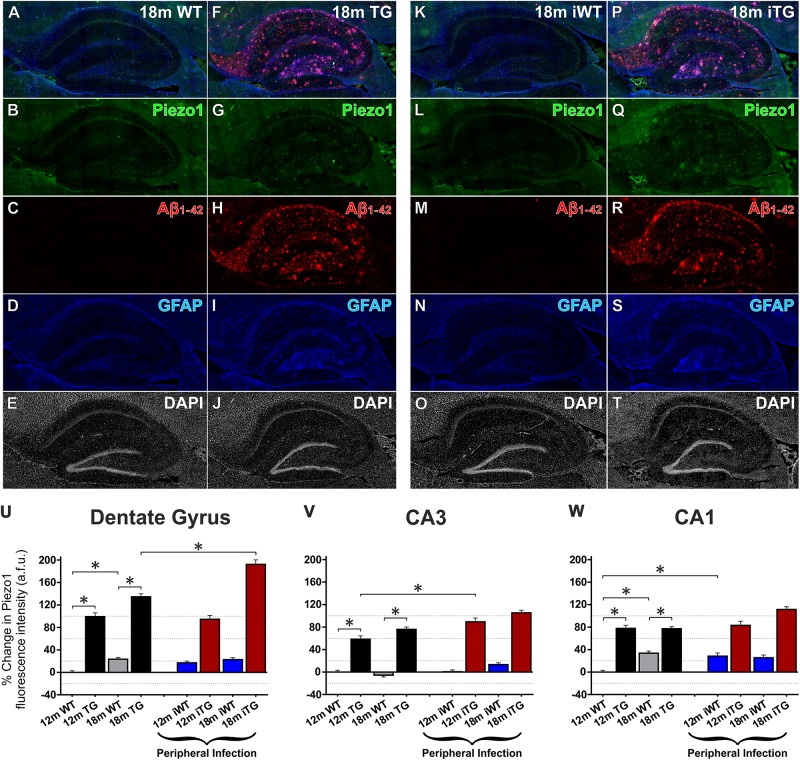
Aging, Aβ_1-42_ pathology and peripheral infection upregulate Piezo1 expression in the hippocampus of TgF344-AD rats. Sagittal sections (lateral -1.9 mm) of WT Fisher and TgF344-AD rat brains were immunofluorescently stained for Piezo1 (N-terminal antibody), conformation-specific Aβ_1-42_, GFAP, and DAPI. Shown are representative images of the hippocampus of an 18-month wild-type (18m WT) rat **(A–E)**, an 18-month TgF344-AD 18m TG **(F–J)**, an 18-month WT with infection (18m iWT) **(K–O)**, and an 18-month TgF344-AD with infection (18m iTG) **(P–T)**. TgF344-AD rats with and without an infection show clear upregulations of GFAP **(I,S)** in hippocampal areas with dense amyloid deposition **(H,R)**, particularly the hilus, the outer molecular layer of the dentate gyrus, and the stratum oriens of the CA1. Moreover, amyloid plaques surrounded by a dense meshwork of GFAP-positive astrocytes generally showed concomitant upregulations of Piezo1 expression, particularly in 18m TG rats with or without an infection **(G,Q)**. Next, changes in Piezo1 expression with age, infection and genotype were quantified in the neuronal cell body layers of the dentate gyrus **(U)**, CA3 **(V)**, and CA1 **(W)**. Piezo1 expression increased from 12- to 18-months in WT rats in the DG and CA1, but not CA3 neurons. A much more potent trigger of Piezo1 expression in the hippocampus, however, was genotype with TgF344-AD rats expressing much larger levels of Piezo1 in the DG **(U)**, CA3 **(V)**, and CA1 **(W)** at 12-months of age. There were no further increases in Piezo1 expression in 18-month old TgF344-AD rats. However, peripheral infection caused a significant increase in Piezo1 expression in the dentate gyrus **(U)** of TgF344-AD rats (18m iTG). Data were normalized to 12m WT values for each hippocampal region and are represented as the mean ± SEM of the percentage change from the 12m WT groups. Three-way ANOVAs with Holm-Sidak *post hoc* tests were performed to test for interactions between age, genotype, and peripheral infection. ^∗^Represents a change of > 20% and a *p*-value < 0.01. There were 5–6 rats per group and 2–3 brain sections per animal. Ten ROI were analyzed per hippocampal region per section, i.e., 100–180 ROIs analyzed per group.

Just as normal aging increased expression of Piezo1 channels in the prefrontal cortex, increases in Piezo1 were also evident in the CA1 (34 ± 4%) and DG (24 ± 3%) of the hippocampus, but not in the CA3 region, from 12 to 18 months in WT rats (Figures 6A–W). Notably, there were also large relative increases in Piezo1 expression in the DG (99 ± 7%; Figure 6U), CA3 (58 ± 6%; Figure 6V) and CA1 (78 ± 6%; Figure 6W) of 12-month TG rats compared to their 12-month WT conspecifics. Interestingly, however, there were no further significant increases in hippocampal Piezo1 expression in TG rats from 12 to 18 months of age, suggesting that amyloid deposition and plaque load begin in the hippocampus before spreading further to cortical regions.

### Peripheral Infection Upregulates Hippocampal Piezo1 Expression in Wild-Type and TgF344-AD Rats

Unlike the cerebral cortex, upregulations in Piezo1 expression were measured in the hippocampal CA1 region (28 ± 5%) in response to peripheral *E. coli* infection at 12 months of age in iWT rats (Figure 6W). However, no further increases in Piezo1 expression were evident in the CA1 of infected 18-month iWT rats. Peripheral infection at 8 and 11 months did, however, cause an upregulation of Piezo1 channels in the CA3 region of iTG rats at 12 months of age (20 ± 7%; Figure 6V). Moreover, repeated peripheral infection at 8, 11, 14, and 17 months of age caused a large increase in Piezo1 channels in the dentate gyrus of 18-month iTG rats (25 ± 8%) compared to their non-infected and age-matched TG counterparts (Figure 6U).

### Piezo1 Expression Strongly Correlates With Amyloid Plaque Deposition in the Hippocampal Dentate Gyrus

**FIGURE 7:**
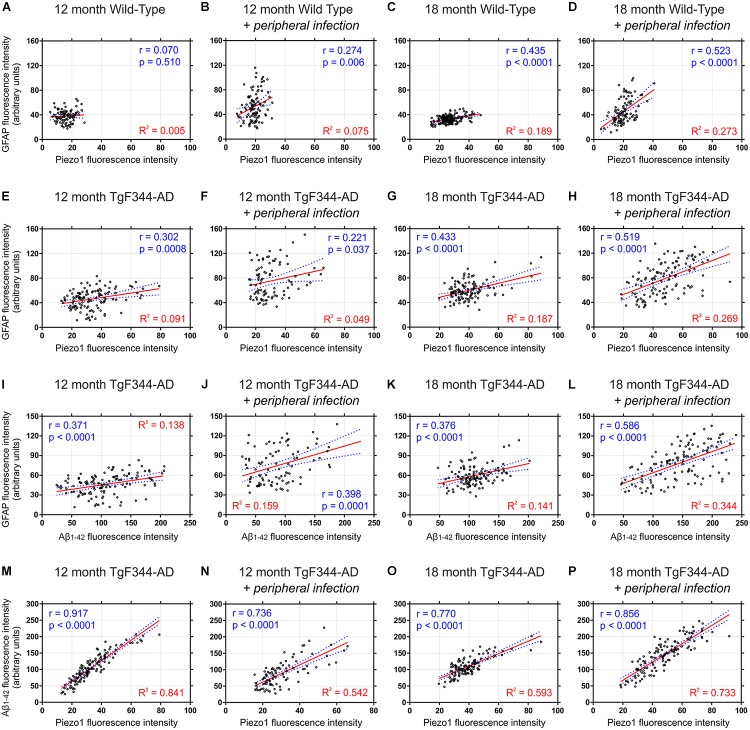
Piezo1 expression in the hippocampal dentate gyrus correlates with GFAP in aged rats with peripheral infection and strongly correlates with amyloid plaques in TgF344-AD rats. Sagittal brain sections were triple-stained for Piezo1, GFAP and conformation-specific Aβ_1-42_ and Pearson correlations (*r* values) were performed to measure changes in Piezo1 channel expression in astrocytes (GFAP vs. Piezo1) with age and peripheral infection in WT **(A–D)** and TgF344-AD **(E–H)** rats. There was a moderate Pearson correlation between GFAP and Piezo1 in the infected 18-month WT (*r* = 0.523) and infected 18-month TgF344-AD rats (*r* = 0.519). However, the linear regression (*R*^2^) values were relatively weak for both groups suggesting that the GFAP fluorescence intensity is not a good predictor of Piezo1 channel expression. Next, Pearson correlations were performed to measure changes in GFAP expression around amyloid plaques (GFAP vs. Aβ_1-42_) in the dentate gyrus with age and peripheral infection in TgF344-AD rats **(I–L)**. There was a moderate Pearson correlation between GFAP and Aβ_1-42_ in the infected 18-month old TgF344-AD rats (*r* = 0.586) but a relatively weak linear regression (*R*^2^) value suggesting that Aβ_1-42_ fluorescence intensity is not a good predictor of GFAP expression. Finally, Pearson correlations were performed to measure changes in Piezo1 channel expression in and around amyloid plaques (Aβ_1-42_ vs. Piezo1) in the dentate gyrus with age and peripheral infection in TgF344-AD rats **(M–P)**. There were strong Pearson correlations between Aβ_1-42_ and Piezo1 in 12-month old TgF344-AD rats (*r* = 0.917), 12-month old TgF344-AD rats with peripheral infection (*r* = 0.736), 18-month old TgF344-AD rats (*r* = 0.770), and 18-month old TgF344-AD rats with peripheral infection (*r* = 0.856). In addition, the high linear regression (*R*^2^) values for each group **(M–P)** suggests that Aβ_1-42_ fluorescence intensity is a good predictor of Piezo1 channel expression in the dentate gyrus of TgF344-AD rats.

It is clear from our immunofluorescence data that both GFAP reactivity and Piezo1 expression are greater around amyloid plaques, especially in the hippocampus. Apart from the molecular layer of the cerebellum, the dentate gyrus displayed the lowest levels of Piezo1 expression in 12-month old WT rats (Figure 2O). Because basal levels of Piezo1 in the DG are low, the relative (%) increases in Piezo1 protein expression in 12-month old, 18-month old, and 18-month infected Tg-344AD rats were large (Figure 6U). To assess the relationship between Piezo1 expression and amyloid plaque pathology in adult and aged rats with and without repeated peripheral infections, we correlated Piezo1 fluorescence intensity with both GFAP and Aβ_1-42_ expression in the dentate gyrus of all groups (Figure 7). In agreement with our *in vitro* studies, Piezo1 expression did not correlate with GFAP expression in 12-month WT, 18-month WT, or 12-month iWT (Figures 7A–C). However, DG Piezo1 expression showed a moderate correlation with GFAP intensity in 18-month iWT rats exposed to repeated peripheral infections (Figure 7D; Pearson *r* = 0.523, *p* < 0.0001). Similarly, there was no correlation between GFAP fluorescence intensity and Piezo1 expression in the DG of 12-month TG, 18-month TG, or 12-month iTG (Figures 7E–G). In 18-month iTG, however, increases in GFAP reactivity in the DG did correlate with increased Piezo1 expression (Figure 7H; Pearson *r* = 0.519, *p* < 0.0001). Peripheral infection of older rats led to an upregulation of GFAP in the hippocampal DG and these reactive astrocytes were more likely to express Piezo1 channels. GFAP expression in the DG of older iTG rats that had repeated peripheral infections also correlated with high amyloid plaque pathology (Figure 7L; Pearson *r* = 0.586, *p* < 0.0001), when compared to 18-month TG rats without infection (Figure 7K). Finally, Piezo1 expression in the DG displayed very strong correlations with Aβ_1-42_ fluorescence intensity in 12- and 18-month TgF344-AD rats either with or without peripheral infection (Figures 7M–P). This suggests that whilst peripheral *E. coli* infection alone can upregulate astrocytic Piezo1; Aβ_1-42_ CMM and *in vivo* amyloid plaque pathology are stronger triggers of mechanosensing Piezo1 channel expression in reactive astrocytes.

## Discussion

TgF344-AD transgenic rats overexpress mutated human PSEN1 (PSEN1ΔE9) and human APP with the Swedish mutation (APP_SWE_) and were developed with the intention of creating an improved animal model of AD that recapitulates a broader spectrum of neuropathological features seen in the human AD brain (Cohen et al., 2013). As such, TgF344-AD rats display neuronal tau deposition in contrast to what is generally observed in transgenic mice with these particular gene mutations (Duyckaerts et al., 2008; Stancu et al., 2014). Unless tau-associated genes are also mutated, transgenic mice tend not to spontaneously form neurofibrillary tau tangles (Holcomb et al., 1998; Jankowsky et al., 2001). By combining this improved transgenic rat model with a UTI, a common comorbidity of elderly people who develop AD, the model we present here more closely mimics the systemic inflammation and the spatiotemporal deposition of amyloid plaques observed in the hippocampus and cortex of aging human AD subjects.

Utilizing the TgF344-AD rat model (Cohen et al., 2013), we show here that Piezo1 protein expression increases in several key brain structures in response to aging, peripheral infection and amyloid plaque pathology. Piezo1 channels increase in the prefrontal cortex and the hippocampal CA1 and dentate gyrus with normal aging. These brain regions are particularly important for processing salient sensory information (Barbas and Zikopoulos, 2007; Lester et al., 2017), for learning and memory (Kane and Engle, 2002; Guo et al., 2018) and for higher executive functions (Arnsten and Li, 2005; Suwabe et al., 2017), all of which deteriorate in the elderly (Zimprich and Kurtz, 2013; Thomé et al., 2016; Wiegand et al., 2016). Since an increase in stretch-activated Ca^2+^ channel expression could, in theory, predispose aging neurons to Ca^2+^ dysregulations (Nagarajan et al., 2014), it would be interesting to further investigate if Piezo1 channel expression modulates neuronal Ca^2+^ homeostasis. Intrinsic neuronal properties and synaptic plasticity are intimately linked with tight control of cellular Ca^2+^ concentrations (Catterall et al., 2013; Maggio and Vlachos, 2014) and, therefore, mechanosensitive channels that regulate calcium levels may play an important role in cognition in the aging hippocampus or prefrontal cortex. In this regard, various members of the transient receptor potential (TRP) channel family have been implicated in both mechanosensation (Kwan et al., 2006) and Ca^2+^ dysregulations in neurons and astrocytes in AD (Yamamoto et al., 2007). In the present study, the age-dependent increases in Piezo1 channel expression measured in the prefrontal cortex, CA1 and DG of wild-type rats appeared predominantly neuronal whereas in the TgF344-AD rat model, peripheral infection and amyloid plaque pathology caused an upregulation of Piezo1 predominantly in reactive astrocytes, although concomitant increases in neurons and reactive microglia are also likely to occur. This is interesting because the role of Piezo1 in CNS neurons could be different to its function in astrocytes since neurons constitutively express mechanosensing Piezo1 channels on axonal growth cones and the outer cell membrane, whereas astrocytes exposed to neuroinflammatory stimuli, such as Aβ_1-42_ CMM, upregulate and accumulate Piezo1 channels within the perinuclear compartment. Indeed, in epithelial cell types Piezo1 reportedly co-localizes with the endoplasmic reticulum where it regulates intracellular Ca^2+^ release from stores (McHugh et al., 2010). Moreover, separating the physiological role of Piezo1-mediated extracellular calcium influx from Piezo1-regulated Ca^2+^ release from intracellular stores will reveal subsets of novel mechanotransduction cascades that originate from either outer membrane-spanning Piezo1 channels or from intracellular Ca^2+^ stores (Clapham, 2007). Our results suggest that Piezo1 channels regulate glial mechanosensation and may trigger astrogliosis in the AD brain. Moreover, amyloid plaque-associated microglia and the biochemical factors they release when activated likely play a key role in controlling Piezo1 expression in reactive astrocytes. As such, TgF344-AD rats infected with *E. coli* show enhanced Piezo1 expression in the cortex and hippocampus compared to non-infected transgenic animals. Moreover, microglia have been shown to regulate the pathogenic activities of astrocytes in a mouse model of multiple sclerosis (Rothhammer et al., 2018). Therefore, determining whether Piezo1 regulates neurodegenerative or neuroprotective mechanotransduction signaling in reactive astrocytes surrounding amyloid plaques is an important topic for future research.

### Astrocytic Piezo1 in Alzheimer’s Disease

Satoh et al. (2006) discovered that astrocytes surrounding amyloid plaques in the cerebral cortex of AD patients express hMib mRNA. Coste et al. (2010) renamed Mib (also known as FAM38A), Piezo1, and described how it forms a stretch-activated calcium-permeable channel in the neuroblastoma cell line, Neuro2A. Elegant new research has revealed the tertiary structure of Piezo1 at near atomic level (Ge et al., 2015) and describes how non-selective cationic channels are formed from homotrimers which exhibit large mechanosensitive ‘propeller-like’ structures on the outer surface of membranes (Coste et al., 2012; Li et al., 2014). Astrocytes are very sensitive to changes in their local mechanical environment (Leipzig and Shoichet, 2009; Blumenthal et al., 2014) and our data suggests that perturbations caused by stiff amyloid plaques trigger the transcriptional upregulation of mechanosensing Piezo1 channels in astrocytes which are normally devoid of Piezo1 protein (Figure 3E). But why might a sub-population of reactive astrocytes upregulate mechanosensing Piezo1 channels? Interestingly, Liddelow et al. (2017) showed that ∼60% of GFAP-positive astrocytes in the prefrontal cortex of human AD patients were of the A1 phenotype, i.e., a neurotoxic subtype of reactive astrocyte that drives neuroinflammation and causes neurotoxicity. It would be interesting to determine if the 30–40% of Piezo1-expressing astrocytes around amyloid plaques in the TgF344-AD rat model stain positive for A1 astrocyte markers (e.g., Serping1), or for astrocytes of the A2 phenotype (e.g., S100A10) which reportedly release neurotrophic factors and are mainly neuroprotective (Liddelow et al., 2017). More research is needed to determine what makes Piezo1-expressing astrocytes around amyloid plaques different to their Piezo1-negative neighbors and to characterize both subpopulations based on their ‘neuroinflammatory phenotype.’

### Piezo1 as a Regulator of Neuron/Glial Communication

Blumenthal et al. (2014) suggest that neuronal Piezo1 is important for sensing the topography of cells at the nanometre scale. Hippocampal neurons cultured *in vitro* form many neuron/glial interactions when grown on relatively smooth glass substrates but blocking mechanosensitive channels with GsMTx4, a peptide component of the *Grammostola rosea* tarantula venom, promotes decoupling of neurons from astrocytes and leads to fewer cell contacts. This suggests that Piezo1 can regulate the physical communication between neurons and astrocytes. Moreover, they also showed that the surface topography of brain tissue becomes rougher in response to amyloid plaque pathology which can increase the nanoroughness of astrocytes, thus contributing to neurodegeneration (Blumenthal et al., 2014). If Piezo1 does indeed regulate neuron/glial communication *in vivo*, this supports our previous work which revealed that Piezo1 channels are involved in neuronal mechanosensation and axon guidance during brain development (Koser et al., 2016), enabling migrating growth cones to detect changes in the stiffness of their local environment. Here, we confirm that the rat optic tract also expresses relatively high levels of Piezo1 channels (Figure 1A), as well as the corpus callosum and cerebellar arbor vitae, suggesting that Piezo1 may play a more general role in axonal pathfinding during brain development. Indeed, migrating axons and growth cones are highly mechano-responsive and likely receive constant feedback about the stiffness of the local ECM or the apparent density of glial cell networks as they grow through different brain regions toward their final targets. Moreover, glial cells are softer than neurons (Lu et al., 2006) and recent studies have shown that SH-SY5Y cells (a human neuroblastoma cell line) that overexpress mutated APP_SWE_ are stiffer than normal SH-SY5Y cells (Lu et al., 2017). Therefore, astrocytes capable of engulfing and phagocytosing neurotoxic Aβ_1-42_ peptides may change their mechanical properties or surface topography in the AD brain. Neurons in contact with reactive astrocytes that engulf neurotoxic amyloid peptides may be able to sense a change in their cellular stiffness or membrane nanoroughness and detach from them. Therefore, widespread loss of cell contacts in the hippocampus and cortex, or a disruption to neuron/glial communication caused by changes in the mechano-responsiveness of cells, may accelerate neurodegeneration (Blumenthal et al., 2014) and cognitive decline in the AD brain.

Recent evidence also suggests that dysregulated mechanosensitive signaling can contribute to astrogliopathy in Alexander disease, leading to an increase in brain stiffness (Wang et al., 2018). Alexander disease is a rare neonatal neurodegenerative disorder caused by accumulation of pathogenic Rosenthal fibers due to mutations in astrocytic GFAP. In contrast, we have recently shown that glial scars rich in GFAP- and Vimentin-expressing glial cells, which form following cortical stab injury or contusive spinal cord injury, are much softer than surrounding healthy CNS tissue (Moeendarbary et al., 2017). Therefore, astrogliosis can apparently lead to either increases or decreases in brain tissue stiffness depending on the underlying causes of tissue injury or disease. As such, reactive astrogliosis can be classified as isomorphic (conserved morphology) or anisomorphic (astrocyte hypertrophy and proliferation) (Verkhratsky et al., 2013). These reactive states differ, not only in morphology, but also in their (patho)physiological functions. For instance, isomorphic gliosis is thought to aid regeneration of neuronal networks by providing a permissive biochemical microenvironment for growth cones to migrate through on their journey to relocate their correct synaptic targets. On the other hand, anisomorphic astrogliosis contributes to glial scar formation which is initially beneficial because it prevents extensive neurodegeneration, but as the primary trauma heals, the glial scar can also inhibit axonal re-growth across the lesion site due to secretion of factors such as chondroitin sulfate proteoglycans (Bradbury et al., 2002; Fawcett, 2006). Interestingly, we have shown that addition of chondroitin sulfate to the *Xenopus* brain *in vivo* causes widespread softening of brain tissue (Koser et al., 2016). Therefore, differences in the secretome of phenotypically and functionally distinct types of reactive astrocyte could, in theory, explain the dichotomy in brain tissue stiffness observed in Alexander disease (Wang et al., 2018) versus cortical glial scars caused by a traumatic stab injury (Moeendarbary et al., 2017). In both cases, however, astrogliosis will likely disrupt neuronal mechanobiological signaling and may trigger either neurodegenerative or neuroprotective/pro-regenerative mechanisms. An important area for future research will involve characterizing the role of Piezo1 in neurons and glial cells in the injured or degenerating brain. With this aim in mind, Piezo1 channel opening reportedly regulates the Hippo signaling pathway (Pathak et al., 2014), which controls cell proliferation and apoptosis. Piezo1 knockdown also decreases levels of the mechanoresponsive transcriptional coactivator, Yes-associated protein (YAP), in neural stem cells (Pathak et al., 2014) suggesting that Piezo1 activation triggers YAP transcriptional activity. Notably, YAP is highly expressed by astrocytes and deletion of the YAP gene induces astrogliosis (Huang et al., 2016). In Alexander disease, astrocytes overexpress nuclear YAP, potentially as a result of increased brain tissue stiffness (Wang et al., 2018). Whether upregulation of YAP is detrimental to astrocytes or induces a protective phenotype is not yet known. YAP has some protective functions and can activate suppressor of cytokine signaling (SOCS) gene expression and, therefore, can prevent astrocyte reactivity by downregulating the JAK–STAT inflammatory pathway (Huang et al., 2016). Taken together, the upregulation of astrocytic Piezo1 that we see around amyloid plaques in the hippocampus and cortex of TgF344-AD rats could, on the one hand, contribute to AD-associated astrogliopathy and neurodegeneration but, on the contrary, astrocytic Piezo1 may instead drive anti-inflammatory signaling cascades through YAP-mediated suppression of cytokine signaling that work in concert to reduce reactive astrogliosis and neuroinflammation in the aging AD brain.

## Conclusion

The data presented here suggests that mechanoresponsive astrocytes surrounding stiff amyloid plaques in the AD brain upregulate Piezo1 channels for, as yet, unknown reasons. Moreover, peripheral infection, a common comorbidity in elderly people with AD, augments Piezo1 channel expression in astrocytes. This suggests a potential role for pro-inflammatory microglia in regulating astrocytic Piezo1 levels. Future studies will focus on the signaling cascades triggered by Piezo1-mediated currents in reactive astrocytes and Piezo1 as a potential therapeutic target for amyloid plaque-induced neurodegeneration in AD.

## Author Contributions

GS and KD conceived the project and designed the research. MV-E, MM, HB, AC, EM, and GS performed the experiments. MV-E, KD, and GS analyzed the data. PW, AS, and EB performed the infections in WT and TG rats. MV-E and GS wrote the manuscript with contributions from all authors. All authors discussed the data and results.

## Conflict of Interest Statement

Evotec (UK) Ltd. was involved in the design of the urinary tract infection study performed on wild-type and TgF344-AD rats and analyzed the urine samples for bacterial load. Evotec (UK) Ltd. was not involved in the design or analysis of any other experiments. The authors declare that the research was conducted in the absence of any commercial or financial relationships that could be construed as a potential conflict of interest.

## Footnotes

1 http://fiji.sc/Fiji

2 http://www.uniprot.org/

## Abbreviations

Aβ42: amyloid-β 1–42 peptide
BSA: bovine serum albumin
CMM: conditioned microglial media
CNS: central nervous system
DMEM: Dulbecco’s modified Eagle’s medium
FBS: fetal bovine serum
GFAP: glial fibrillary acidic protein
HBSS: Hank’s buffered salt solution
HRP: horse radish peroxidase
iTG: transgenic with infection
iWT: wild-type with infection
NaCl: sodium chloride
NFH: neurofilament H
PBS: phosphate-buffered saline
PVDF: polyvinylidene difluoride membrane
SDS: sodium dodecylsulfate
TG: transgenic
TNF-α: tumor necrosis factor-alpha
WT: wild-type
